# Chemical Fingerprinting and Antimicrobial Potential of Selected Ethnomedicinal Plants: Correlation Between Quercetin Content and Bioactivity

**DOI:** 10.3390/plants15121915

**Published:** 2026-06-20

**Authors:** Felicia Dragan, Daria Marina Dragan, Alexandra Cristina Tocai (Moțoc), Andrei George Teodorescu, Melinda Haydee Kovacs, Emoke Dalma Kovacs, Cristina Mihali, Camelia Daniela Ionaș, Alina Claudia Groze

**Affiliations:** 1 Department of Pharmacy, Faculty of Medicine and Pharmacy, University of Oradea, 410028 Oradea, Romania; farmafeli@gmail.com; 2 Faculty of Medicine and Pharmacy, Student of the University of Oradea, 410073 Oradea, Romania; 3 Department of Preclinical Disciplines, Faculty of Medicine and Pharmacy, University of Oradea, 410073 Oradea, Romania; 4 Department of Morphological Disciplines, Faculty of Medicine and Pharmacy, University of Oradea, 410073 Oradea, Romania; teodorescu_andrei_george@yahoo.com; 5 Research Institute for Analytical Instrumentation, National Institute for Research and Development in Optoelectronics INOE 2000, 67 Donath Street, 400293 Cluj-Napoca, Romania; melinda.kovacs@icia.ro (M.H.K.); dalma.kovacs@icia.ro (E.D.K.); 6 Department of Chemistry and Biology, Faculty of Sciences, Technical University of Cluj-Napoca, 76 Victoriei Street, 430122 Baia Mare, Romania; mihali.cristina@gmail.com; 7 Department of Chemistry, Faculty of Computer Science and Sciences, University of Oradea, 410087 Oradea, Romania; ticaratdaniela@yahoo.com (C.D.I.); acozma@uoradea.ro (A.C.G.)

**Keywords:** medicinal plants, HS-SPME GC–MS, quercetin quantification, antimicrobial activity, principal component analysis

## Abstract

Due to their diverse phytochemical composition, medicinal plants belonging to the families Amaryllidaceae, Lamiaceae, and Myrtaceae possess antimicrobial and antioxidant properties. In this study, six ethanolic extracts of *Allium ursinum*, *Allium sativum*, *Allium cepa*, *Salvia rosmarinus*, *Ocimum basilicum*, and *Syzygium aromaticum* were analyzed by HS-SPME GC-MS and HPLC. Their chemical composition was evaluated and compared by chemometrics and their biological activity determined by an antimicrobial assay. A total of 72 compounds was detected (terpenoids, phenolic derivatives, fatty acids, and phytosterols). In *Allium* species, phytosterols were mainly abundant, whereas *O. basilicum* extracts were characterized by high contents of linalool and *S. rosmarinus* by 2-hydroxychalcone and 4-hydroxybutanoic acid lactone. Principal component analysis distinguished chemically species-specific chemical profiles, whilst the HPLC evaluation resulted in the highest quercetin content in *S. rosmarinus* extracts, which also displayed the best antibacterial effect against *Staphylococcus aureus*. Despite the observed correlation between the quercetin content and antibacterial activity, no definitive relation could be established without biological replicates, MIC evaluation, and tests with isolated compounds.

## 1. Introduction

Medicinal plants are a valuable source of bioactive phytochemicals such as antioxidants and antimicrobials like phenolics, terpenoids, flavonoids, and phytosterols [1]. Over the past decade, the prevalence of resistant bacteria against conventional antibiotics has led to increased research on natural products that can inhibit harmful bacteria while minimizing the toxicity caused by artificial antibiotics. Secondary metabolites from medicinal plants have garnered much attention from researchers due to their interference with cellular processes and oxidative mechanisms in microbes [2].

Species classified under the families Amaryllidaceae, Lamiaceae, and Myrtaceae are renowned for their abundance in phytochemicals and medicinal uses. Examples include *Allium cepa*, *Allium sativum*, *Allium ursinum*, *Salvia rosmarinus*, *Ocimum basilicum*, and *Syzygium aromaticum*, which have been found to possess several types of phytochemical compounds such as sulfur compounds, terpenes, flavonoids, phenolic acids, and essential oils, which may be responsible for their antimicrobial and antioxidant properties [3,4,5,6,7,8]. Most of the research carried out on these plants is individual-based, and they mostly concentrate on either phytochemistry or biological activity.

The latest analytical methods like HS-SPME GC–MS and HPLC are important for the analysis and quantification of phytochemicals, while chemometric techniques, including PCA and hierarchical clustering, are important for differentiating between species through phytochemical fingerprinting.

Despite numerous studies on medicinal plants, comparative investigations integrating phytochemical profiling, quercetin quantification, chemometric analysis, and antimicrobial evaluation across multiple ethnomedicinal species under a unified experimental design remain limited. Thus, the objective of this research is to conduct a comparative analysis on the phytochemical content and the antimicrobial properties of ethanolic extracts derived from the following medicinal plants: *Allium cepa* L., *Allium sativum* L., *Allium ursinum* L., *Salvia rosmarinus* Spenn., *Ocimum basilicum* L., and *Syzygium aromaticum* L. The methods used for the study include HS-SPME GC-MS analysis, quercetin measurement by HPLC, hierarchical clustering, PCA, and antimicrobial assays against *Staphylococcus aureus*, *Escherichia coli*, and *Pseudomonas aeruginosa*.

## 2. Results

### 2.1. Morphologic Aspects of Ethnomedicinal Plants

The selected medicinal plants were chosen for their previous reports of ethnomedicinal significance, their antimicrobial activities and antioxidant potential. These plants belong to different botanical families, are known to have rich sources of different bioactive phytocompounds, and therefore were appropriate for their comparison of phytochemical and antimicrobial activities.

The botanical classification and ethnomedicinal importance together with important phytochemical constituents of medicinal plants examined were presented in Table 1.

### 2.2. Phytochemical Profiling by GC–MS Analysis

GC-MS showed clear interspecific variations in their phytochemical content. These differences in chemical contents most likely could be responsible for the different antibacterial activities and are likely the reasons for the species-specific clustering detected by chemometric analysis.

Tentative identification based on NIST (National Institute of Standards and Technology) library matching suggested the presence of a complex phytochemical profile in the investigated plant extracts, with significant qualitative and quantitative differences between species. A total of 72 compounds were identified, predominantly belonging to terpenoids, phenolic derivatives, fatty acids, and phytosterols (Table 2). The dominant compounds in *A. ursinum* were Stigmasterol (35.447%), Campesterol (31.174%) and Hexacosanol (15.833%), while in *A. sativum* they were Campesterol (33.363%), Stigmasterol (26.705%) and α-Linolenic acid (4.411%). The major compound identified in *A. cepa* was Isoboldine (30.040%), followed by Campesterol (13.197%) and Longifolenaldehyde (11.313%). Most importantly, superior phytochemical profile of the *A. ursinum* leaves were obtained from Oradea, Romania, compared to the *A. cepa* bulb and *A. sativum* bulb from commercial sources. In *S. aromaticum*, the most abundant compound was p-Isopropylphenetole (42.795%), while in *O. basilicum* it was linalool (33.872%), and in *S. rosmarinus* it was 2-Hydroxychalcone (25.432%) and 4-Hydroxybutanoic acid lactone (22.123%).

#### 2.2.1. Chemical Classification of Identified Compounds

**Figure 1:**
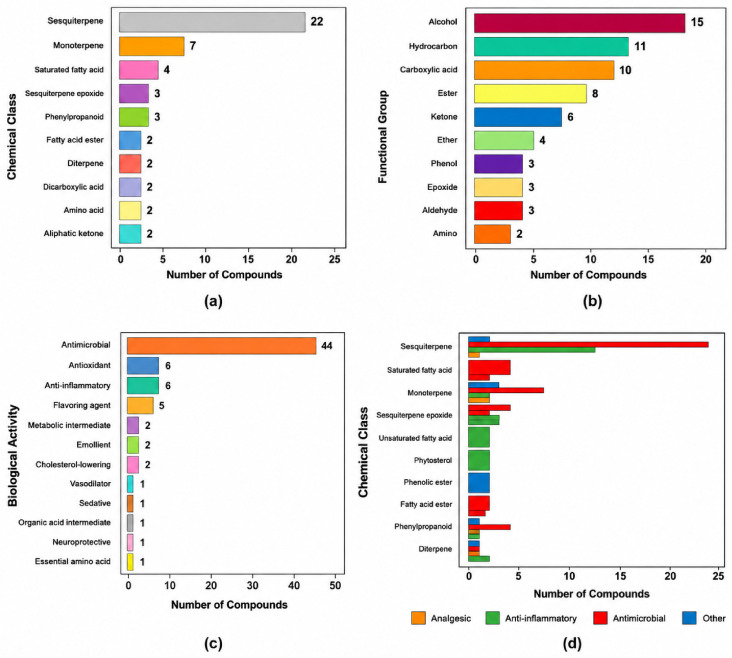
Identified compounds classification. (**a**) Chemical class distribution; (**b**) functional groups distribution; (**c**) biological activities distribution; (**d**) biological activities by chemical class.

A total of 72 compounds were detected by GC–MS, representing 31 unique chemical classes (Figure 1a). The distribution of compounds across chemical classes was characterized using frequency counts and percentages. Sesquiterpenes were the most represented class (*n* = 21, 29.17%), followed by monoterpenes (*n* = 7, 9.72%) and saturated fatty acids (*n* = 4, 5.56%). The top 10 chemical classes accounted for 66.67% of all identified compounds. Among the detected compounds, fifteen distinct functional groups were identified (Figure 1b). The most prevalent functional groups were alcohols (*n* = 15, 20.83%), hydrocarbons (*n* = 11, 15.28%), and carboxylic acids *(n* = 10, 13.89%). The top 10 functional groups represented 90.28% of all compounds, demonstrating concentration in major functional categories. Between these compounds, twelve unique biological activities were documented (Figure 1c). The distribution of biological activities was significantly non-uniform (χ^2^ = 269.67, df = 11, *p* < 0.001), with antimicrobial activity being predominant (*n* = 44, 61.11%), followed by anti-inflammatory (*n* = 6, 8.33%) and antioxidant activities (*n* = 6, 8.33%). This skewed distribution reflects the strong antimicrobial potential of the analyzed compounds. The relationship between chemical classes and biological activities was examined for classes containing ≥2 compounds (*n* = 13 classes). Figure 1d shows that monoterpenes exhibited the broadest activity spectrum, with antimicrobial (*n* = 7), antioxidant (*n* = 3), anti-inflammatory (*n* = 2), and analgesic (*n* = 2) properties. Due to sparse data and small cell counts, Fisher’s exact test was deemed more appropriate than chi-square test for assessing independence between chemical class and activity type.

#### 2.2.2. Compounds Differentiation Among Samples

**Figure 2:**
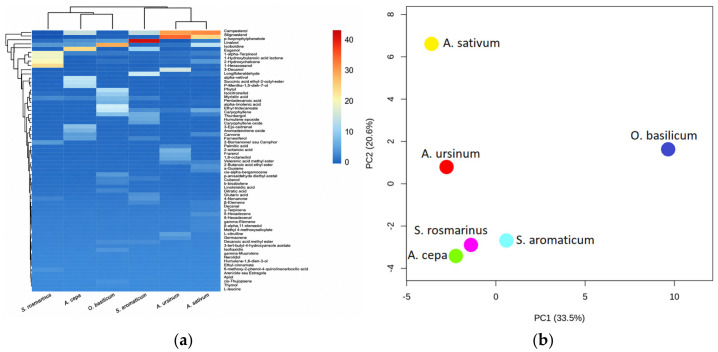
Compound distribution among samples. (**a**) Heatmap of compound distribution; (**b**) PCA plot of sample clustering based on compound profiles.

The hierarchical clustering heatmap revealed distinct chemical fingerprints across the six plant extracts, with clear differentiation in compound composition profiles (Figure 2a). The analysis of compounds included clustering performed using Euclidean distance and complete linkage methods for both samples (columns) and compounds (rows). The heatmap color gradient (blue to red) illustrated concentration ranges from 0% to >40%. Several compounds showed sample-specific accumulation patterns. Stigmasterol and campesterol exhibited strong signals exclusively in *A. ursinum* leaves and *A. sativum* bulb samples, appearing as red clusters in the heatmap. Conversely, p-isoprophylphenetole was uniquely abundant in *S. aromaticum* flower buds (42.8%), while linalool showed high concentrations in *O. basilicum* leaves extract (33.9%) and moderate levels in *A. sativum* bulb (12.3%). The vertical clustering of compounds revealed functional groups with similar distribution patterns across samples. Terpene compounds (linalool, L-alpha-terpineol, p-mentha-1.5-dien-7-ol) clustered together, showing preferential accumulation in *O. basilicum* leaves extract and *S. rosmarinus* leaves. Sterol compounds formed a distinct cluster, predominantly present in *A. ursinum* leaves and *A. sativum* bulb. The majority of compounds (approximately 60%) showed low concentrations (<5%) across all samples, appearing as blue regions in the heatmap. *O. basilicum* leaves extract exhibited the highest chemical diversity with 38 detectable compounds, followed by *A. ursinum* leaves (31 compounds), while *A. cepa* bulb extract showed the lowest diversity with only 12 compounds. Principal component analysis was performed to assess the overall variance in chemical composition among the six plant extracts and to visualize their relationships in multivariate space. The first two principal components (PC1 and PC2) collectively explained 54.0% of the total variance (PC1: 33.5%, PC2: 20.6%), indicating substantial chemical differentiation among samples (Figure 2b). The PCA score plot suggested separation of the six analyzed samples, with no overlapping positions in the PC1–PC2 space, suggesting distinct chemical fingerprints for each extract. *O. basilicum* leaves extract exhibited the most pronounced separation, positioned at the extreme positive end of PC1 (score: +9.60), suggesting a unique chemical profile dominated by volatile terpene compounds, particularly linalool (33.9%). This marked separation along PC1 indicates that the primary source of variance in the dataset is driven by terpene content and related volatile compounds. *A. ursinum* leaves and *A. sativum* bulb, both members of the *A.* genus, showed moderate proximity along PC1 (scores: −2.78 and −3.65, respectively) but were clearly differentiated along PC2. *A. sativum* bulb occupied the most positive position on PC2 (+6.58), while *A. ursinum* leaves were positioned near the origin (+0.79). *A. cepa* bulb extract, *S. aromaticum* flower buds, and *S. rosmarinus* leaves formed a loose cluster in the negative quadrant of both PC1 and PC2 (PC1 range: −2.26 to +0.52; PC2 range: −3.42 to −2.66). *S. aromaticum* flower buds showed slight positive displacement along PC1 (+0.52), consistent with its high concentration of p-isoprophylphenetole (42.8%).

### 2.3. HPLC Quercetin Content

There was great variation in the content of quercetin within the analyzed extracts (Table 3).

The concentration of quercetin in *S. rosmarinus* leaves is approximately 19 times higher than in *A. cepa* bulb and 6 times higher than in *S. aromaticum* buds. *A. sativum* bulb has a higher concentration of quercetin (1.46 ± 0.05 mg/100 g) than *A. cepa* bulb (1.01 ± 0.03 mg/100 g). *A. ursinum* has the lowest value out of this set (0.92 ± 0.01), suggesting that its therapeutic benefits may be based on sulfur compounds or vitamin C, rather than quercetin.

### 2.4. Correlation Between Quercetin Content and Antimicrobial Activity

Pearson correlation coefficient analysis showed that there is a high positive correlation between the amount of quercetin and the inhibitory effect on Gram-positive bacteria *Staphylococcus aureus* (r = 0.925, *p* = 0.008), which may indicate a potential contribution of quercetin to antibacterial activity. However, no correlations were found between quercetin and inhibition zones for Gram-negative bacteria *Escherichia coli* (r = 0.650, *p* = 0.162) and *Pseudomonas aeruginosa* (r = 0.124, *p* = 0.815). The comprehensive statistical relationships among those bacterial strains were presented in Table 4.

To assess the strength of correlations using a non-parametric test, Spearman rank correlation coefficient analysis was conducted. It showed that the relationship between quercetin and *S. aureus* inhibition zone was positively correlated but not statistically significant (ρ = 0.714, *p* = 0.136). No statistically significant correlations were observed.

The difference in results between Pearson and Spearman analyses may be explained by the small sample size (*n* = 6) and the high concentration of quercetin obtained from *S. rosmarinus* extract.

**Figure 3:**
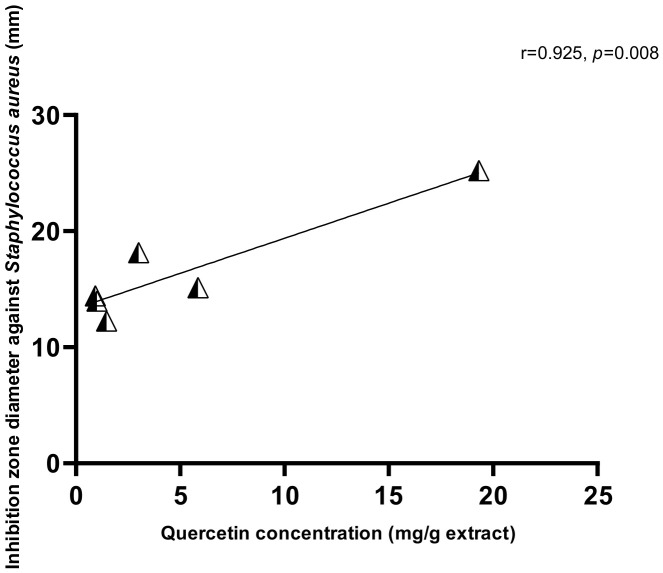
Correlation between quercetin concentration and the inhibition zone diameter against *Staphylococcus aureus*.

The Pearson correlation coefficient analysis showed a very high positive correlation between the level of quercetin and its inhibitory activity against *Staphylococcus aureus* (r = 0.925, *p* = 0.008). This is shown in Figure 3. However, no significant correlation was found between the level of quercetin and its inhibitory activity against Gram-negative bacteria such as *Escherichia coli* (r = 0.650, *p* = 0.162) and *Pseudomonas aeruginosa* (r = 0.124, *p* = 0.815).

### 2.5. Antimicrobial Activity of Medicinal Plant Extracts

**Figure 4:**
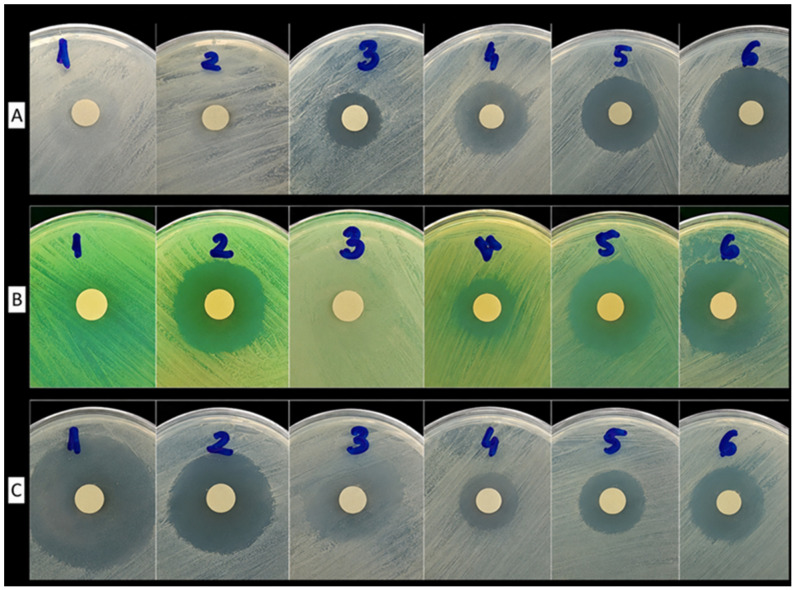
Representative inhibition zones produced by ethanolic extracts of six medicinal plants against (**A**) *Escherichia coli*, (**B**) *Pseudomonas aeruginosa*, and (**C**) *Staphylococcus aureus;* 1—*A. sativum*, 2—*O. basilicum*, 3—*A. cepa*, 4—*A. ursinum*, 5—*S. rosmarinus*, 6—*S. aromaticum.* Images are representative of three independent experiments (*n* = 3).

The formation of inhibition zones confirmed the antibacterial potential of the tested extracts (Figure 4).

Sterile paper disks (6 mm) impregnated with plant extracts were placed on Mueller–Hinton agar plates inoculated with standardized bacterial suspensions (0.5 McFarland). After 24 h incubation at 37 °C, inhibition zones were measured in millimeters using a digital caliper. Images show representative plates from three independent experiments (*n* = 3).

Table 5 summarizes antimicrobial activities of medicinal plant extracts tested against some Gram positive and Gram negative bacteria.

Among the tested extracts, *S. rosmarinus* leaves showed the highest antibacterial activity, particularly against *S. aureus* (25.21 ± 0.39 mm), showing a statistically significant difference (*p* < 0.05) compared to the five medicinal plant extracts (Table 4).

## 3. Discussion

Significant qualitative variations were observed among the volatile profiles of the six medicinal plant extracts based on HS-SPME GC-MS analyses. PCA and hierarchical clustering established that each of the extracts had a distinct phytochemical fingerprint. Clusters were consistent with botanical relationship and the main class of metabolites present in each species. The Lamiaceae species, *O. basilicum* and *S. rosmarinus*, showed higher levels of terpenoids. Higher percentages of phytosterols, campesterol and stigmasterol, were obtained from *Allium* species. These results are in accordance with those of many other publications on the description of Lamiaceae plants as having abundant volatile terpenoids and *Allium* species as a rich source of sterols and sulfur compounds [3,4,17,19,20,24,25].

Another important result obtained by the HS-SPME GC-MS analysis was the volatile profile of *S. aromaticum*, with p-isopropylphenetole being the most abundant compound detected (42.80% RPA). This finding contrasts with most of the literature stating that eugenol is the main component of *S. aromaticum* essential oil, usually present in amounts of 70–90% of the total volatile fraction, followed by eugenyl acetate and -caryophyllene [31].

There may be several reasons for this discrepancy. First, the present study employed HS-SPME GC–MS, which selectively samples volatile compounds from the headspace above the sample, whereas most published studies have analyzed essential oils obtained by hydrodistillation or steam distillation. As mentioned above, all of these methods extract specific fractions of plant metabolites. Hence, chemical profiles detected are anticipated to be diverse. Second, *S. aromaticum* volatile composition is also likely to be dependent on the geographical region, the season of collection and harvesting time, storage time, and processing of the postharvest clove. The above-mentioned parameters have been known to cause qualitative and quantitative changes in the volatile constituents of plant tissues.

The high amount of p-isopropylphenetole observed in the current study shows how responsive HS-SPME GC-MS is to the variations in headspace, and that the plant material analyzed here has a specific volatile fingerprint different from that typically reported in traditional essential-oil research. However, additional experiments with triplicate samples and additional extraction methods would need to be performed in order to show whether this is indeed a chemotype or simply due to the extraction method used. The HPLC analysis clearly showed differences in the amount of quercetin in all the samples. *S. rosmarinus* has by far the most amount of quercetin, followed by *O. basilicum* and then *S. aromaticum*, although levels in the *Allium* spp. are much lower. Such differences are consistent with previous reports showing that flavonoid accumulation is strongly influenced by species-specific metabolic pathways as well as environmental and agronomic factors [3,19,24]. Nevertheless, because *A. ursinum* was collected from a natural population, whereas the remaining plant materials were commercially sourced, part of the observed variation may also reflect differences in geographical origin, harvesting season, processing, and storage conditions. A further limitation of this method is that, despite the reduced pressure at 35 °C for solvent removal, certain heat-sensitive constituents are still susceptible to degradation.

Correlation analysis revealed a strong positive Pearson association between quercetin concentration and inhibition of *Staphylococcus aureus*. The corresponding Spearman correlation was not significant. This is probably due to small sample size and the high concentration of quercetin in *S. rosmarinus* extract which gave a very different result. These findings should be interpreted as indicating a possible relationship rather than proving one.

In addition, the antibacterial effects are not likely to be mediated by the action of quercetin alone since most plant extracts contain a lot of active compounds and synergism is common. Further works like testing the pure compound and testing using minimum inhibitory concentrations should be conducted to determine whether the various compounds found in the plant extracts act on each other and whether quercetin itself contributes to antibacterial activity.

Of all the extracts studied *S. rosmarinus* possessed the highest level of antibacterial activity; this was most evident against *Staphylococcus aureus*. The reasons for the higher susceptibility of *S. aureus* compared to any of the tested Gram-negative bacteria might lie in differences between Gram-negative and positive cell envelopes. Gram-negative bacteria have an additional outer membrane that hinders passage of hydrophobic phytochemicals, such as terpenoids and phenolics, through the cell envelope. Gram-positive bacteria are more readily able to transport such molecules through their cell envelope. Although the diffusimetric method is an effective screening tool for the evaluation of antimicrobial potential, it has certain limitations related to the differential diffusion capacity of phytochemical compounds in solid culture media [32]. The hydrophobic nature of certain secondary metabolites (e.g., terpenoids from *S. rosmarinus*) may underestimate the real biological activity [33]. In addition, a blank sterile paper-disk control was not included in this study as the commercially sterilized discs are normally biologically inert. Nevertheless, including such a control could have further strengthened the experimental design by excluding the paper matrix’s potential contribution, and it should be considered in future investigations.

This pronounced activity from *S. rosmarinus* may be partially linked to its elevated quercetin content (19.31 ± 0.06 mg/g); although the analysis on hand does not establish a direct causal link between this and the observed activity. It has been reported that quercetin has antimicrobial effects through a variety of mechanisms, including membrane permeabilization, inhibition of nucleic acid synthesis, and disruption of bacterial enzyme systems [34]. Moreover, it has also been observed to potentiate the action of antibiotics such as tetracycline, doxycycline, ciprofloxacin and gentamycin to improve cell lysis, reduce biofilm matrix components, and disrupt the *S. aureus* cell membrane [35]. In addition to quercetin, the high number of terpenoids within *S. rosmarinus* may be expected to provide a range of other activities through a similar process. Quercetin derivatives have been established as important flavonoids in *A. cepa* and play a significant role in the antioxidant and antibacterial activities of the plant, but the concentrations of these compounds identified in this work were lower than in *S. rosmarinus*, thus potentially accounting for the lower interaction with antibacterial activities.

Observations of chemometrics and correlations should therefore be considered exploratory and hypothesis-generating. In order to confirm the observed associations, biological and technical replications will be required, as well as quantitative antimicrobial assays, such as MIC determinations, and targeted testing of purified compounds.

## 4. Materials and Methods

### 4.1. Plant Material

All plant material used included six ethanolic preparations with an identical plant:solvent ratio of 1:10 (*w/v*). The plant material of *A. ursinum* used in the study was collected from Oradea, Romania. A specimen of the species was kept in the herbarium of the Faculty of Medicine and Pharmacy Oradea, Romania, registered in NYBG Steere Herbarium, Uop 05 730. Immediately after harvest, leaves of *A. ursinum* were dried separately at room temperature and ground with an electric mill with a fast-rotating knife. The extract was obtained by maceration in ethanol 70. The shredded leaves of *A. ursinum* (15 g) were placed in a cartridge, which was left to macerate for 10 days. The extraction of bioactive compounds was carried out with 150 mL of absolute ethanol (99%). The solvent was removed under reduced pressure at 35 °C using a Büchi R-300 rotary evaporator (BüCHI Labortechnik AG, Flawil, Switzerland).

The other five plants (*A. cepa*, *A. sativum*, *S. rosmarinus*, *O. basilicum*, and *S. aromaticum*) were purchased from a local herbal supplier and authenticated morphologically. Drying, grinding, and sieving of the plant materials were carried out to obtain the homogeneous powder. For each plant, maceration was performed by immersing 15 g of plant material in 150 mL of ethanol (70) for ten days at room temperature without exposure to light. Filtration of the solutions was followed by solvent removal at 35 °C under reduced pressure using Büchi R-300 rotatory evaporator. The same plant-to-solvent ratio (1:10, *w/v*) and extraction conditions were applied for all extracts to ensure comparability among samples.

### 4.2. HS-SPME GC-MS Analysis

HS-SPME GC-MS (Headspace Solid-Phase Microextraction coupled with Gas Chromatography–Mass analysis) was applied for raw extracts organic compounds profile analysis using the method described by Dippong et al., 2022 [36]. Briefly, 5 mL of the sample was transferred to a 20 mL headspace vial. The headspace vials were sealed with crimp-top caps with TFE-silicone headspace septa (Thermo Fischer Scientific, Waltham, MA, USA). Each vial was incubated for 20 min at 60 °C. Afterward, the SPME fiber Divinylbenene/Carboxen/Polydimethylsiloxane (50 µm DVB/30 µm CAR/30 µm PDMS) was exposed for 15 min (60 °C) at the headspace of the sample to perform the HS-SPME extraction of volatile organic compounds. Furthermore, the extracted volatile organic compounds were desorbed for 7 min from the fiber coating into the Thermo Fischer Scientific Trace 1310 GC gas chromatograph injection port set at 250 °C. The volatile organic compounds were separated using a DB-WAX capillary column (30 m × 0.25 mm i.d. × 0.25 µm film thickness, J&W Scientific Inc. (Folsom, CA, USA)). Ultrahigh-purity helium was used as a carrier gas at a linear velocity of 1 mL/min. The oven temperature program was as follows: initial temperature of 35 °C, heated to 180 °C at a rate of 5 °C·min^−1^, increased to 230 °C at a rate of 15 °C·min^−1^, and then held at a plateau for 7 min. Mass spectra were recorded in electron impact (EI) ionization mode at 70 eV using a TSQ 9000 MS, Thermo Fischer Scientific mass spectrometer. The quadrupole mass detector, ion source, and transfer line temperatures were set at 150, 230 and 280 °C, respectively. Mass spectra were scanned in the range *m*/*z* 50–450 amu. Detected compounds were identified by comparing the mass spectra with the NIST 14 database system library and linear retention index. The criteria for compound identification required a mass spectrum matching score of ≥80%. The results were reported as %RPA where values of peak area for each identified compound is divided by the sum of the peak area for all identified compounds; these relative values indicate the relative abundance, rather than absolute concentration, since there was no determination of compound-specific response factors. These values reflect relative abundance and are not a true quantitative representation of the absolute concentration of a compound due to the fact that specific response factors for compounds were not measured. The total ion chromatogram (TIC) of each sample was used for peak area integration.

### 4.3. HPLC Quercetin Determination

The analysis was performed using high-performance liquid chromatography (HPLC) with reversed-phase chromatography. A YL Instrument 9100 HPLC system (Young Lin Instrument Co., Ltd., 2016, now YOUNGIN Chromass, Hogye-dong, Anyang, South Korea) was used, consisting of a vacuum solvent degassing system, a quaternary pump for four solvents, a UV–VIS detector, and a Zorbax Eclipse XDB-C18 analytical column (250 × 4.6 mm, particle size 5 μm), Agilent Technologies, Santa Clara, CA, USA packed with octadecylsilane-bonded silica gel. A guard column (12.5 × 4.6 mm) with the same stationary phase was used.

The chromatographic method for the analysis of quercetin was adapted from Ang et al., 2014 [37]. The operating conditions were as follows: isocratic elution with a mobile phase consisting of 60% 2% (*v*/*v*) aqueous acetic acid solution (prepared using ultrapure water with a resistivity of 18.2 MΩ·cm produced by a DIRECT-Q3 UV ultrapure water purification system, Millipore Merck KGaA, Darmstadt, Germany and 40% acetonitrile; flow rate of 1.3 mL/min; UV detection at 370 nm; column oven temperature maintained at 35 °C; and injection volume of 20 μL using a Hamilton syringe Hamilton Company, Reno, NV, USA.

Before injection, plant extract samples were diluted with methanol (dilution factor 5) and filtered through 0.45 μm Millipore membrane filters.

Total analysis time was 15 min. The HPLC system was controlled by a microprocessor using YL-Clarity software version 8.1, which allowed for chromatogram acquisition and data processing.

Before analyzing the plant extracts, the calibration curve was drawn with standard solutions of quercetin of concentrations between 0.5 and 80 mg/L (0.5; 1; 5; 10; 20; 30; 40; 50; 60 and 80 mg/L). The standard solutions of concentrations 0.5–60 mg/L were obtained by successive dilutions of the primary standard solution of 80 mg/L. Quercetin reagent from Aldrich Fluka, Taufkirchen, Germany was used to prepare the primary standard solution.

A calibration curve was obtained with the equation: A = 121.76554 × C_quercetin_ with the correlation coefficient r = 0.99954. Based on this calibration curve, the chromatograph YL-Clarity software version 8.1 displayed the quercetin concentration.

### 4.4. Antimicrobial Activity Assay

#### 4.4.1. Preparation of Bacterial Strain

A streak of tested bacteria slant was mixed in 5 mL of nutrient broth and then incubated at 30 °C. The inoculum was adjusted to 0.5 McFarland standard (approximately 1.5 × 10^8^ CFU/mL). Antibacterial activity was evaluated using the following reference strains: *Staphylococcus aureus* subsp. aureus (WDCM 00034, ATCC 25923; lot no. 360-661-8, expiration 07/2026, in use from 1 November 2025), *Escherichia coli* (WDCM 00013, ATCC 25922; lot no. 335-588-41, expiration 08/2026, in use from 1 October 2025), and *Pseudomonas aeruginosa* (WDCM 00025, ATCC 27853; lot no. 353-558-61, expiration 06/2026, in use from 0=5 November 2025). All strains were obtained from Microbiologics World Headquarters (Minnesota, USA) and were handled according to the manufacturer’s instructions.

#### 4.4.2. Disc Diffusion Method

The antibacterial activity of *A. cepa*, *A. sativum*, *A. ursinum*, *O. basilicum*, *S. rosmarinus*, and *S. aromaticum* ethanolic extracts were estimated by the disk diffusion method. All extracts were prepared by the same method in relation to plant material and solvent (1:10, *w*/*v*). Dried extracts for antibacterial assay were reconstituted with 5% DMSO at a concentration of 10 mg/mL. Sterilized paper disks of 6 mm diameter were impregnated with 20 µL of each extract solution (i.e., 0.2 mg plant extract/disc). The same concentration of extracts and amount of extracts/disks was used for all treatments [38]. The saturated disks (6 mm) were placed on the surface of Mueller–Hinton agar (MHA) plates previously inoculated with standardized bacterial suspension. The disks saturated with Ciprofloxacin were positive controls. Discs impregnated with 5% DMSO were used as negative controls and showed no inhibition zones. The MHA plates were incubated at 37 °C for 24 h. The inhibition zones (mm) around disks were measured by a ruler. The final concentration of DMSO did not exceed 5% (*v*/*v*) and was confirmed to not inhibit bacterial growth.

#### 4.4.3. Statistically Analysis

Samples from each medicinal plant were analyzed, and all assays were performed in triplicate. The data of analysis are represented as mean value ± standard deviation (SD). The data were subjected to analysis by one-way ANOVA (Tukey’s multiple comparison test) at *p* < 0.05 significant level. To evaluate the relationship between the concentration of bioactive compounds (quercetin) and the antimicrobial activity (inhibition zones), Pearson and Spearman correlation coefficients were calculated. All statistical analyses were performed using GraphPad Prism software (version 8.0.2).

## 5. Conclusions

Six medicinal plant extracts belonging to the families Amaryllidaceae, Lamiaceae, and Myrtaceae were investigated for their phytochemical diversity. HS-SPME GC-MS revealed chemically unique fingerprints of species, whereas multivariate analyses allowed us to distinguish the different plant species by their volatile profiles. Among the tested plant extracts, *S. rosmarinus* presented the highest concentration of quercetin, which displayed a significant activity on *Staphylococcus aureus*, as indicated by disk diffusion method. Furthermore, a significant positive correlation was evidenced between the concentrations of quercetin in plant extracts and antibacterial activity. Overall, further studies such as experiments with MIC determinations and biological replicates of the bioactivity tests, including in vitro determination of biological activity and study of isolated phytochemicals for elucidation of synergy, should be performed to further clarify this possible relationship and investigate the contribution of each single phytochemical and possible interactions between them.

## Figures and Tables

**Table 1:** Ethnomedicinal relevance and major bioactive constituents of the investigated plant species.

Family	Scientific/Common Name	Part Used	Geographical Distribution	Global Presence	Main Phytochemicals	Biological Properties	GBIF Data Source (DOI)
Amaryllidaceae	*Allium cepa* L./Onion	Bulb	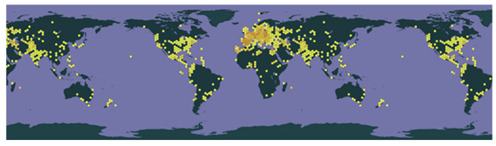	Worldwide; major producers include China, India, and the USA [9]	Quercetin, organosulfur compounds	Cardioprotective, hypoglycemic, hypolipidemic, stomachic, antibacterial, antihelmintic, antiviral, antioxidant, and immunomodulatory properties [9,10]	https://doi.org/10.15468/dl.m4m9sf (accessed on 25 May 2026) [11].
Amaryllidaceae	*Allium sativum* L./Garlic	Bulb (Cloves)	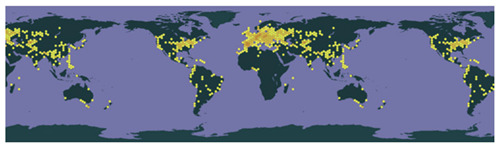	Naturalized in temperate regions globally [12]	Allicin, sulfur compounds	Antioxidant, antibacterial, antiviral, hepatoprotective, anti-inflammatory, cardioprotective, anticancer, and antidiabetic effects [12,13]	https://doi.org/10.15468/dl.yqqqee (accessed on 25 May 2026) [14].
Amaryllidaceae	*Allium ursinum* L./Wild Garlic	Leaves	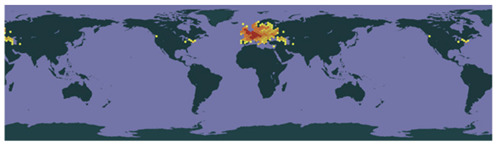	In European forests [15]	Sulfur compounds, flavonoids	Antibacterial, antioxidant, antidiarrheal, antiphlogistic, and antitumor properties [16,17]	https://doi.org/10.15468/dl.et5cv2 (accessed on 25 May 2026) [18].
Lamiaceae	*Salvia rosmarinus* Spenn./ Rosemary	Leaves	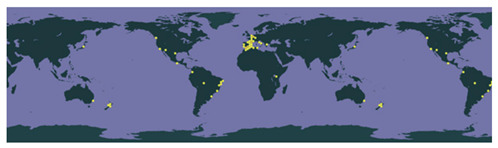	Cultivated globally in temperate and subtropical climates [19]	Rosmarinic acid, carnosic acid	Antifungal, antibacterial, antioxidant, analgesic, anti-inflammatory, antirheumatic, antispasmodic in renal colic and dysmenorrhoea, carminative and choleretic activities [20,21]	https://doi.org/10.15468/dl.m2vhnu (accessed on 25 May 2026) [22].
Lamiaceae	*Ocimum basilicum* L./Sweet basil	Leaves	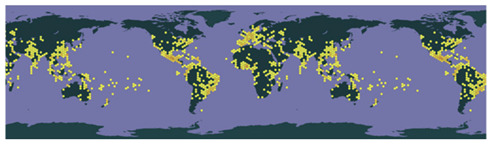	Widely cultivated in Mediterranean, Asian, and American regions [23]	Linalool, estragole	Antimicrobial, antiviral, antifungal, antioxidant, antispasmodic, anti-inflammatory, antidiabetic, antitumor, and atherosclerotic properties [24,25]	https://doi.org/10.15468/dl.sxbxtb (accessed on 25 May 2026) [26].
Myrtaceae	*Syzygium aromaticum* L./Clove	Flower buds	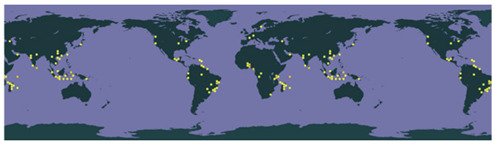	Cultivated in tropical maritime climates (Madagascar, Zanzibar, Sri Lanka) [27]	Eugenol	Antibacterial, antiviral, antifungic activities, hypoglycemic, antitumor, and anti-inflammatory effects [28,29]	https://doi.org/10.15468/dl.crqh27 (accessed on 25 May 2026) [30].

All images with maps of the geographical distribution of each plant were generated using the GBIF network; specific data download DOIs for each plant are provided in Table 1.

**Table 2:** Comparative chemical composition of the analyzed medicinal plant extracts.

Compounds	*A. ursinum* Leaves	*A. sativum* Bulb	*A. cepa* Bulb	*S. aromaticum* Flower Buds	*O. basilicum* Leaves	*S. rosmarinus* Leaves
Eugenol	0.614	1.315	2.390	0	1.014	17.888
Linalool	0.489	12.333	4.804	0	33.872	4.086
4-Hydroxybutanoic acid lactone	0.489	3.784	0	0	0.600	22.123
Isopinocamphone	0.888	0	0	0.232	0	0
Frarrerol	0.930	1.407	0	0	0	0
2-Bornanone	3.148	0	0	0	0	0
L-alpha-Terpineol	0.293	0	0	0	0	16.118
p-Mentha-1.5-dien-7-ol	0.140	0	0	0.092	7.835	0
2-Hydroxychalcone	0.059	0	0	0	0	25.432
d-Glucoheptose	0	0.718	0	0	0	0
Carvone	0.259	0.169	0	0	0	3.744
2-Butanoic acid ethyl ester	0.239	0	0	0	1.906	0
4-Nonanone	0	0.813	0	0	0	0
L-citrulline	0	0	0	0	0.085	0
Anethole	0.168	0	0	0	0.356	1.459
Thymol	0.379	0.710	0	0	0	0.821
Citraconic acid	0	0.762	0	0	0	0
p-Isoprophylphenetole	0	0	0	42.795	2.910	0
L-leucine	0	1.515	0	0	0.050	1.002
a-Guaiene	0	0	0	0.469	1.092	0.332
Carophyllene	0	0	0	4.227	0.221	0
cis-alpha-bergamotene	0	0	0	0	1.114	0
Aromandrene	0	0	0	0.568	0.482	0.254
Germacrene	0	0	0	0	0.075	0
Ethyl-cinnamate	0.063	0	0	0	0.335	0
Methyl 4-methoxysalicylate	0.039	0	0	0	0	0
Isoledene	0	0	0	0.338	0.243	0
gamma-Muurolene	0	0	0	0	0.446	0
Decanoic acid methyl ester	0.058	0	0	0	0.300	0.440
Isofraxidin	0.085	0.233	0	0	0.388	0
gamma-Elemene	0	0	0	0.275	0.616	0
p-anisaldehyde diethyl acetal	0.104	0	0	0	0.793	0
Nerolidol	0	0	0	0.115	0.451	0
3-tert-butyl-4-hydroxyanisole acetate	0.152	0.178	0	0	0.214	0.294
6-methoxy-2-phenol-4-quinolinecarboxilic acid	0.052	0	0	0	0.302	0
Humulane-1.6-dien-3-ol	0	0	0	0	0.221	0
Diffractaic acid	0.043	0.553	0	0.463	0.190	0
b-bisabolene	0	0	0	1.098	0.257	0
b-humulene	0.445	0	0	0	0.112	0
2-Octanone	0	0.749	0	0	0.054	0
Cubenol	0	0	0	0	0.913	0
Apiol	0	0	0	0	0	1.529
p-Eudesmol	0	0.865	0	0	0	0
Isocitronellol	0.189	0	1.539	1.276	5.367	1.652
cis-Thujopsene	0	0	0	0	0	1.642
Glutaric acid	0.292	0.512	0	0	0	0
Humulene epoxide	0	0	5.615	0	0	0
alpha-vetivol	0	0	10.310	0	0	0
3-Epi-cedrenal	0	1.367	2.972	0	0	0
Longifolenaldehyde	0	0	11.313	0	0	0
1.8-octanediol	0	2.215	0	0	0	0
3-Decanol	0	0.533	0	13.337	0	0
Charophyllene oxide	0	0	4.918	0	0	0
8-alpha,11-elemadiol	0	0.312	0	0	0	0
Aromandrene oxide	0	0	3.120	1.211	0	0
Valerenic acid methyl ester	0	1.658	0	0	0	0
Palmitic acid	2.770	0	0	0	0	0
Cedrol	0	0.667	0	0	0	0
Pentadecanoic acid	0	0	0	0	10.763	0
Ethyl tridecanoate	0	1.296	0	4.346	4.958	0
Myristic acid	0	0.855	0	0	4.958	0
Phytol	0	0	0	0	6.307	0.556
Linoelaidic acid	0	0	0	1.081	0	0.630
alpha-linolenic acid	0.734	4.411	0	2.894	9.167	0
1-Hexacosanol	15.833	0	0	0	0	0
Farnesilferol	1.645	0	0	0	0	0
Thunbergol	0	0	0	3.172	0.700	0
Succinic acid ethyl-2-octyl-ester	0	0	9.783	0	0.333	0
Campesterol	31.174	33.363	13.197	12.428	0	0
Isoboldine	0	0	30.040	9.582	0	0
2-octanoic acid	2.780	0	0	0	0	0
Stigmasterol	35.447	26.705	0	0	0	0

**Table 3:** HPLC analysis results for quercetin content in plant extracts.

No.	Sample	t_R_ (min)	C_extract_ (mg/g) × 100
1	*A. cepa* bulb	7.232	1.01 ± 0.03 ^a^
2	*A. sativum* bulb	7.773	1.46 ± 0.05 ^b^
3	*A. ursinum* leaves	7.715	0.92 ± 0.01 ^a^
4	*S. rosmarinus* leaves	7.275	19.31 ± 0.06 ^e^
5	*O. basilicum* leaves	7.132	5.85 ± 0.01 ^d^
6	*S. aromaticum* flower buds	7.657	3.00 ± 0.03 ^c^

t_R_—Retention Time; C_extract_—concentration of quercetin in the plant subjected to extraction; lowercase letters mean significant differences (*p* < 0.05) between the data in the column.

**Table 4:** Correlation between quercetin concentration and antibacterial activity against different bacterial strains.

Bacterial Strain	Pearson r	Pearson p	Spearman ρ	Spearman p
*S. aureus*	0.925	0.008	0.714	0.136
*E. coli*	0.650	0.162	0.714	0.136
*P. aeruginosa*	0.124	0.815	0.429	0.419

**Table 5:** Antimicrobial activity of tested extracts from medicinal plants.

Positive Control Ciprofloxacin 5 mcg (Bacteria) 28.00 ± 0.50 mm Negative Control DMSO 0.00 ± 0.00 mm	*E. coli*	*S. aureus*	*P. aeruginosa*
*A. cepa* bulb	10.02 ± 0.56 ^cB^	13.98 ± 0.31 ^cA^	9.07 ± 0.34 ^bB^
*A. sativum* bulb	6.06 ± 0.5 ^dB^	12.27 ± 0.66 ^dA^	6.04 ± 0.09 ^cB^
*A. ursinum* leaves	12.41 ± 0.44 ^bB^	14.44 ± 0.39 ^cA^	8.03 ± 0.36 ^bC^
*S. rosmarinus* leaves	20.11 ± 0.37 ^aB^	25.21 ± 0.39 ^aA^	8.91 ± 0.26 ^bC^
*O. basilicum* leaves	13.03 ± 0.49 ^bB^	15.13 ± 0.34 ^cA^	13.16 ± 0.47 ^aB^
*S. aromaticum* flower buds	18.99 ± 0.22 ^aA^	18.16 ± 0.23 ^bA^	8.07 ± 0.56 ^bB^

Data are expressed as the mean value ± SD (*n* = 3). Lowercase letters mean significant differences (*p* < 0.05) on the column (differences between plant organs and the same microorganism), while the different uppercase letters mean significant differences ((*p* < 0.05) on the line (between the same organ compared to the three investigated bacteria). DMSO—Dimethyl sulfoxide. DMSO was used as negative control and showed no inhibitory effect.

## Data Availability

All datasets generated or analyzed during this study are included in this published article and its Supplementary Information Files. Further inquiries can be directed to the corresponding author.

## Supplementary Materials

The following supporting information can be downloaded at: https://www.mdpi.com/article/10.3390/plants15121915/s1, Figure S1. Morphological appearance of the plant materials used for extraction: (A) bulb of *A. cepa*, (B) bulb of *A. sativum*, (C) leaves of *A. ursinum*, (D) leaves of *S. rosmarinus*, (E) leaves of *O. basilicum*, and (F) flower buds of *S. aromaticum* (personal photos); Table S1. System suitability test parameters for quercetin; Figure S2. Calibration curve of quercetin based on the standard quercetin solutions containing between 0.5 and 80 mg/L; Figure S3. Chromatogram of *Allium cepa* bulb registered at 370 nm; Figure S4. Chromatogram of *Allium sativum* bulb registered at 370 nm; Figure S5. Chromatogram of *Allium ursinum* leaves extract registered at 370 nm; Figure S6. Chromatogram of *Salvia rosmarinus* leaves extract registered at 370 nm; Figure S7. Chromatogram of *Ocimum basilicum* leaves extract registered at 370 nm; Figure S8. Chromatogram of *Syzygium aromaticum* flower buds extract registered at 370 nm.

## Abbreviations

The following abbreviations are used in this manuscript:

DF	Dilution Factor
HS-SPME GC–MS	Headspace Solid-Phase Microextraction coupled with Gas Chromatography–Mass Spectrometry
PCA	Principal Component Analysis
tR	Retention Time
