# Supplementary material for: Chemical Fingerprinting and Antimicrobial Potential of Selected Ethnomedicinal Plants: Correlation Between Quercetin Content and Bioactivity
Source: Plants (Basel). 2026 Jun 20;15(12):1915. doi: 10.3390/plants15121915 (PMC13306324; doi:10.3390/plants15121915)
Supplement: Supplementary file 1 [file plants-15-01915-s001.zip › plants-4367596-supplementary.pdf]

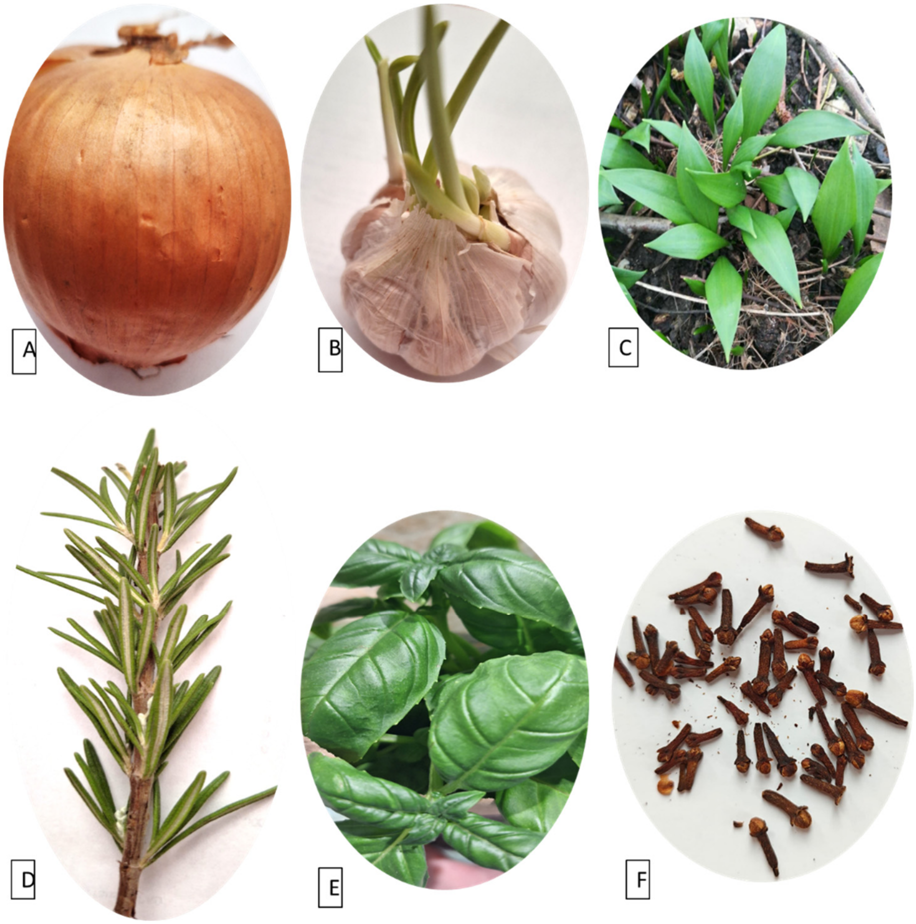

Figure S1. Morphological representation of the plant organs subjected to extraction: (A) *A. cepa* – bulb, *(B) A. sativum* – bulb, *(C) A. ursinum* – leaves, *(D) S. rosmarinus* – leaves, *(E) O. basilicum* – leaves, *(F) S. aromaticum* – fruits (personal photos)

Methodological verification data of Quercetin HPLC analysis

To check the suitability of Quercetin HPLC analysis, a seria of experiments were performed. The system suitability was proved by succesive analysis of quercetin standard solutions of 5 mg/L (5 µg/mL). The retention time, theoretical plates , assimetry values and peak areas values were registered and depicted in Table 1. The low values of relative standard deviations showed a good reproducibility.

Table S1: System suitability test parameters for quercetin

Quercetin standard , 5 mg/L (5 µg/mL), n=6

Parameter	Quercetin (±% RSD)

Retention time (minutes)	7.22±0.068
Theoretical plates	7585.83 ± 0.89
Asymmetry	1.428 ± 0.018
Area (mV·sec)	1241.113 ± 1.68

The calibration curve was obtained

A calibration curve of quercetin based on the standard quercetin solutions containing between 0.5 and 80 mg/L was drawn (Figure S2).

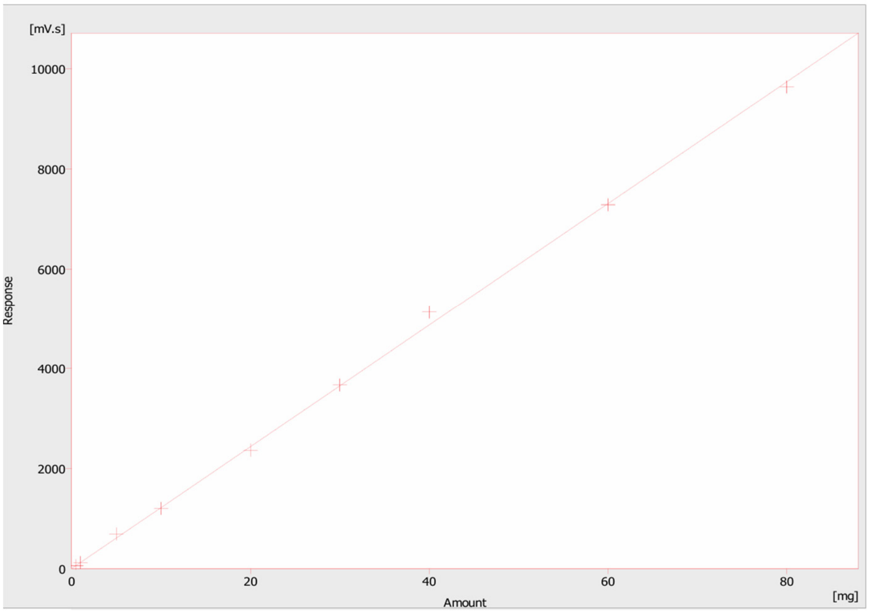

Figure S2. Calibration curve of quercetin based on the standard quercetin solutions containing between 0.5 and 80 mg/L

The calibration equation was: A_{peak} =121.76554*C, with a good correlation correlation coefficient, R= 0.999542.

Recovery rate and precision

Recovery rate was analysed by injecting standard solutions of 0.8; 2 and 15 mg/L quercetin and determining their concentrations by attaching the calibration curve. The HPLC analysis of quercetin solutions was performed in triplicate. The recovery rates were: 100.12 ±0.8 %; 100.42 ± 0.49 %respective 99.98 ± 1.87 %.

The RSD of mean asssay values ranged between 0.75 and 1.88 % indicating satisfactory precision of the HPLC method of quercetin analysis.

Stability

The intermediate precision study accomplished in different days by different analysis for 10 mg/L standard solution of quercetin showed a good stability with RSD values of 0.71 % respective 1.06%.

Retention times varied between different days and therefore it was necessary to analyze at least one standard solution in each series of measurements.

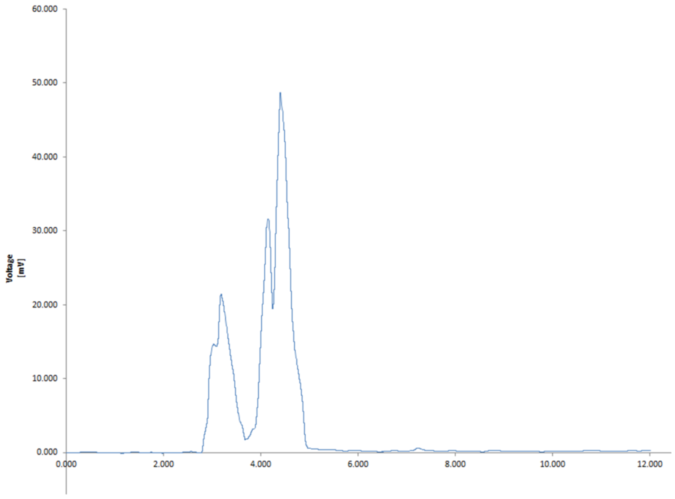

Figure S3. Chromatogram of *Allium cepa* bulb registered at 370 nm. Quercetin was detected at a retention time of 7.232 min and represented 2.1% of the quantified compounds.

	Reten. Time [min]	Response	Amount [mg]	Amount [%]	Peak Type	Compound Name
1	3.015	134.602	0.000	0.0		
2	3.182	442.828	0.000	0.0		
3	4.148	439.767	0.000	0.0		
4	4.398	1043.797	0.000	0.0		
5	7.232	25.391	0.208	2.1	ISTD1	Quercetin
	Total		10.000	2.1		

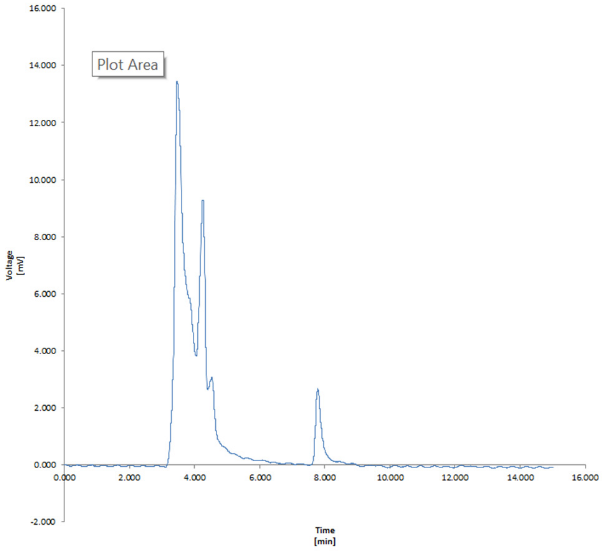

Figure S4. Chromatogram of *Allium sativum* bulb registered at 370 nm.Quercetin was detected at a retention time of 7.773 min and represented 2.8% of the quantified compounds.

	Reten. Time [min]	Response	Amount [mg]	Amount [%]	Peak Type	Compound Name
1	3.457	335.217	0.000	0.0		
2	4.240	133.610	0.000	0.0		
3	4.523	85.068	0.000	0.0		
4	7.773	34.582	0.283	2.8	ISTD1	Quercetin
	Total		10.000	2.8		

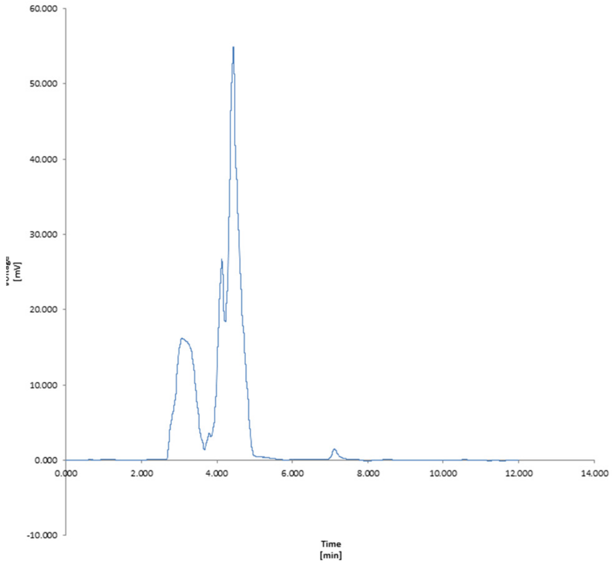

Figure S5. Chromatogram of *Allium ursinum* leaves extract registered at 370 nm. Quercetin was detected at a retention time of 7.715 min and represented 1.8% of the quantified compounds.

	Reten. Time [min]	Response	Amount [mg]	Amount [%]	Peak Type	Compound Name
1	3.415	895.946	0.000	0.0		
2	4.615	239.677	0.000	0.0		
3	5.565	33.202	0.000	0.0		
4	7.715	22.380	0.183	1.8	ISTD1	Quercetin
	Total		10.000	1.8		

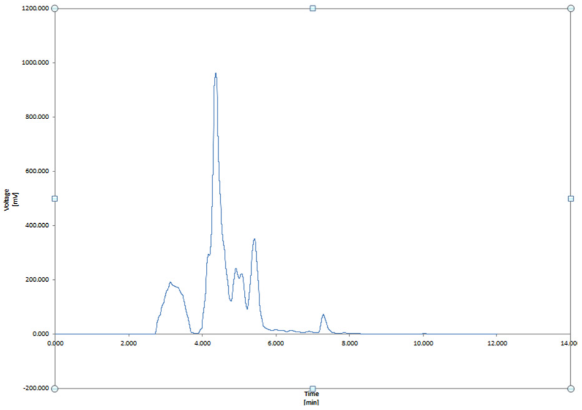

Figure S6. Chromatogram of *Salvia rosmarinus* leaves extract registered at 370 nm. Quercetin was detected at a retention time of 7.275 min and represented 6.9% of the quantified compounds.

	Reten. Time [min]	Response	Amount [mg]	Amount [%]	Peak Type	Compound Name
1	3.125	7142.756	0.000	0.0		
2	4.358	18649.204	0.000	0.0		
3	4.908	2556.580	0.000	0.0		
4	5.058	2373.097	0.000	0.0		
5	5.408	5499.739	0.000	0.0		
6	5.992	177.754	0.000	0.0		
7	6.175	133.176	0.000	0.0		
8	6.392	260.672	0.000	0.0		
9	6.892	143.803	0.000	0.0		
10	7.275	848.666	6.952	69.5	ISTD1	Quercetin
11	7.842	128.795	0.000	0.0		
12	10.025	23.191	0.000	0.0		
13	10.592	15.077	0.000	0.0		
14	11.042	10.059	0.000	0.0		
15	11.258	18.272	0.000	0.0		
16	11.758	15.845	0.000	0.0		
	Total		10.000	69.5		

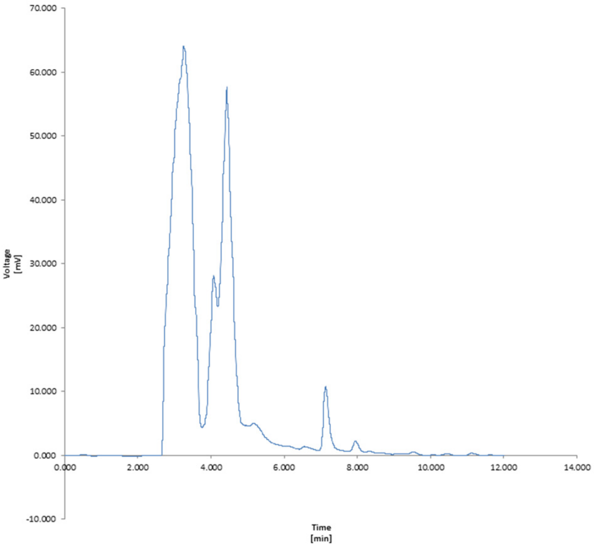

Figure S7. Chromatogram of *Ocimum basilicum* leaves extract registered at 370 nm. Quercetin was detected at a retention time of 7.132 min and represented 0.9% of the quantified compounds.

template: 000000000000 (Channel 1)

	Reten. Time [min]	Response	Amount [mg]	Amount [%]	Peak Type	Compound Name
1	3.248	2506.391	0.000	0.0		
2	4.065	412.688	0.000	0.0		
3	4.432	1232.103	0.000	0.0		
4	5.148	180.191	0.000	0.0		
5	6.532	19.054	0.000	0.0		
6	7.132	114.770	0.940	9.4	ISTD1	Quercetin
	Total		10.000	9.4		

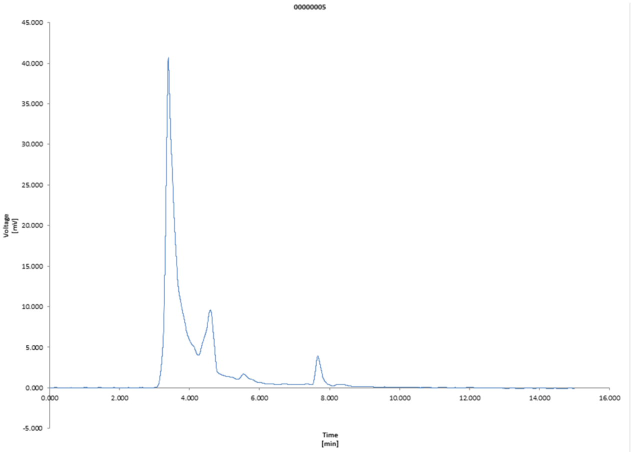

Figure S8. Chromatogram of *Syzygium aromaticum* fruits extract registered at 370 nm. Quercetin was detected at a retention time of 7.657 min and represented 0.6% of the quantified compounds.

	Reten. Time [min]	Response	Amount [mg]	Amount [%]	Peak type	Compound Name
1	3.390	882.602	0.000	0.0		
2	4.590	251.960	0.000	0.0		
3	5.523	65.281	0.000	0.0		
4	7.657	73.717	0.604	6.0	ISTD1	Quercetin
	Total		10.000	6.0		
